# Association between Gastroesophageal Reflux Disease and Appendicitis: A Population-Based Case-Control Study

**DOI:** 10.1038/srep22430

**Published:** 2016-03-02

**Authors:** Li-Ting Kao, Ming-Chieh Tsai, Herng-Ching Lin, Cha-Ze Lee

**Affiliations:** 1 Graduate Institute of Life Science, National Defense Medical Center, Taipei, Taiwan; 2 Sleep Research Center, Taipei Medical University Hospital, Taipei, Taiwan; 3 Division of Gastroenterology, Department of Internal Medicine, Cathay General Hospital, Hsinchu Branch, Taiwan; 4 School of Health Care Administration, Taipei Medical University, Taipei, Taiwan; 5 Department of Internal Medicine, National Taiwan University Hospital, Taipei, Taiwan

## Abstract

Appendicitis and gastroesophageal reflux disease (GERD) are both prevalent diseases and might share similar pathological mechanisms. The aim of this study was to investigate the association between GERD and appendicitis using a large population-based dataset. This study used administrative claims data from the Taiwan Longitudinal Health Insurance Database 2005. We identified 7113 patients with appendicitis as cases, and 28452 matched patients without appendicitis as controls. This study revealed that GERD was found in 359 (5.05%) cases and 728 (2.56%) controls (*p* < 0.001). Conditional logistic regression shows that the adjusted odds ratio (OR) of GERD for cases was 2.05 (95% confidence interval (CI): 1.08~2.33) compared to controls. The adjusted ORs of prior GERD for patients aged 18~39, 40~59, and ≥60 years with appendicitis were 1.96 (95% CI: 1.56~2.47), 2.36 (95% CI: 1.94~2.88), and 1.71 (95% CI: 1.31~2.22) than controls, respectively. We concluded that patients with appendicitis had higher odds of prior GERD than those without appendicitis regardless of age group.

Appendicitis is a widespread abdominal emergency with a lifetime incidence ranging 7~14%^1^,^2^. Annually, 280,000 patients undergo appendectomies to avoid severe complications of appendicitis in the United States^3^,^4^. However, the pathophysiology of appendicitis still remains unclear. Obstruction of the appendiceal lumen is an accepted pathogenesis for acute appendicitis^4^,^5^. Previous studies reported that luminal obstruction can be caused by adhesions, fecaliths, or lymphoid hyperplasia which are frequently due to viral, bacterial, and fungal infections^6^,^7^. Some studies further suggested that a secondary bacterial infection, hygiene, low-fiber diets, and local infection with Fusobacterium spp. etc. are other potential reasons for appendicitis^7^,^8^,^9^. Additionally, spasms and hypertonicity of the neuromusculature at the appendicocecal juncture due to a sympathetic-parasympathetic imbalance were also considered to be possible reasons for appendicitis^10^.

Gastroesophageal reflux disease (GERD) is a prevalent gastrointestinal diagnosis in outpatient clinics^11^,^12^. Approximately 10~20% patients in western countries and 5% patients in Asia experience the symptoms of GERD, such as heartburn and dysphagia^13^. Prior studies suggested that patients with GERD have altered autonomic nervous functions and gastrointestinal motility problems^14^,^15^,^16^. In addition, increasing evidence supports that proton pump inhibitors (PPIs), which are commonly prescribed to patients with GERD, induce hypochlorhydria and further contribute to the overgrowth of some bacteria, including Fusobacterium^17^,^18^,^19^. These underlying mechanisms are believed to be etiologically involved in GERD and appendicitis.

Nevertheless, even though both GERD and appendicitis might share similar pathological mechanisms, no study has ever attempted to explore the association between GERD and appendicitis. Therefore, the purpose of this study was to investigate the association between previously diagnosed GERD and appendicitis using a large population-based dataset in Taiwan.

## Materials and Methods

### Database

This population-based case-control study used administrative claims data from the Taiwan Longitudinal Health Insurance Database 2005 (LHID2005). The LHID2005 includes longitudinal data on medical claims for 1 million individuals since the beginning of the Taiwan National Health Insurance (NHI) program in 1995. These 1 million enrollees were randomly selected from all enrollees involved in the 2005 Registry of Beneficiaries (*n* = 25.68 million) under the NHI program. To date, numerous studies which used data from the Taiwanese NHI program have been published in international peer-reviewed journals^20^,^21^. The LHID2005 consists of de-identified secondary data released to the public for research purposes and was exempted from a full review following consultation with the National Defense Medical Center Institutional Review Board.

### Study Sample

This study design included a study and control group. The study group initially contained 9616 patients who were hospitalized with a principal discharge diagnosis of appendicitis (ICD-9-CM codes 540, 540.0, 540.1, and 540.9) from January 2002 to December 2012. However, in Taiwan, if a hospitalized patients who was suspected of having appendicitis, but was confirmed to have had unqualified appendicitis, his/her discharge diagnosis would be coded as ICD-9-CM code 541 (unqualified appendicitis). Therefore, this study will not include patients with unqualified appendicitis. The date of the first diagnosis of appendicitis was defined as the index date. We then excluded patients under 18 years old (*n* = 2503) in order to limit the study to the adult population. As a result, 7113 patients with appendicitis were included in the study group.

The matched controls (*n* = 28,452) (four controls per patient with appendicitis) were sourced from the residual beneficiaries of the LHID2005. This control group was selected by matching patients with appendicitis in terms of sex, age group (18~29, 30~39, 40~49, 50~59, 60~69, 70~79, and ≥80 years), and year of the index date. For the control group, the year of the index date was simply a matched year in which the controls had a healthcare utilization. In addition, for controls, the date of their first use of ambulatory care during in a matched year was defined as the index date. We assured that none of the selected controls had been diagnosed with appendicitis since the beginning of the NHI program in 1995.

### Outcome measures

In this study, we attempted to find the relationship between GERD and appendicitis. We identified cases with GERD based on ICD-9-CM code 530.11 or 530.81. We only included GERD cases who have received ≥2 GERD diagnoses in order to increase diagnosis validity. In addition, this study only included patients who have received at least one diagnosis of GERD prior to the index date and one GERD diagnosis which was made by a certified gastroenterologist.

### Statistical Analysis

All analyses in this study were performed with the SAS system (SAS System for Windows, vers. 9.2, SAS Institute, Cary, NC). A Chi-squared test was conducted to compare differences in monthly income (<NT$15,841, 15,841~25,000, ≥25,001), geographic location (northern, central, eastern, and southern Taiwan), and urbanization level (5 levels, with 1 being the most urbanized and 5 being the least) between patients with and those without appendicitis. Conditional logistic regressions (stratified on gender, age group, and index year) were used to investigate the association between appendicitis and prior GERD. Additionally, subgroup analyses were conducted to investigate odds ratios (ORs) for GERD of sampled patients by age group (18~39, 40~59, and ≥60 years) because previous literatures reported that the incidence of appendicitis varied among the age groups and age may affect the classic presentation of appendicitis^2^,^22^. The conventional *p* < 0.05 was used to estimate statistical significance in this study.

## Results

The study group included 7113 patients with appendicitis and 28,452 patients in the matched control group. The 35,565 total patients in the study sample had a mean age of 41.3 ± 16.8 years. The demographic characteristics and obese condition of patients with and those without appendicitis are given in Table 1. After matching for gender, age group, and index year, there were significant differences in monthly income (*p* < 0.001) and geographic region (*p* = 0.018) between the study and control groups.

Table 2 exhibits the prevalence, odds ratios (ORs), and 95% confidence intervals (CIs) of prior GERD between cases and controls. It reveals that 1087 (3.06%) of total sampled patients had GERD before the index date. GERD was found in 359 (5.05%) cases and in 728 (2.56%) controls. The conditional logistic regression analysis (stratified by gender, age group, and index year) indicated that the crude OR for prior GERD for cases was 2.04 (95% CI: 1.79~2.30, *p* < 0.001) compared to controls. In addition, the adjusted OR of prior GERD for cases was 2.05 (95% CI: 1.08~2.33, *p* < 0.001) higher than controls after adjusting for monthly income and geographic region.

Table 3 further presents the prevalence of prior GERD between cases and controls according to age group. We found that appendicitis was associated with prior GERD in all age groups. Respective crude ORs of prior GERD for patients aged 18~39, 40~59, and ≥60 years with appendicitis were 1.97 (95% CI: 1.57~2.48), 2.36 (95% CI: 1.94~2.88), and 1.67 (95% CI: 1.29~2.17) compared to controls. Moreover, after adjusting for monthly income and urbanization level, the adjusted ORs for prior GERD for patients aged 18~39, 40~59, and ≥60 years with appendicitis were respectively 1.96 (95% CI: 1.56~2.47), 2.36 (95% CI: 1.94~2.88), and 1.71 (95% CI: 1.31~2.22) higher than the controls.

## Discussion

This population-based case-control study found an association between appendicitis and prior GERD. Results showed that patients with appendicitis were 2.05-times more likely to be diagnosed with prior GERD than those without appendicitis. To the best of our knowledge, no relevant study has ever attempted to investigate the potential relationship between GERD and appendicitis to date, although these two diseases might share analogous pathological pathways.

The actual mechanisms of the association between GERD and appendicitis still remain unclear. The higher odds of prior GERD in patients with appendicitis than those without appendicitis might be explained by an autonomic imbalance, dietary habits, and bacterial infections. The prior literature reported that patients with GERD have a higher prevalence of abnormal autonomic nervous function^14^,^23^. Delayed gastric emptying and gastrointestinal motility problems also frequently occur in patients with GERD^15^,^16^,^23^. In addition, the spasms and hypertonicity of the neuromusculature at the appendicocecal juncture caused by an autonomic imbalance were suggested to be possible explanations for appendicitis^10^. This pathway was found to be etiologically involved in GERD and appendicitis.

As for dietary habits, many studies reported a relationship between low fiber intake and the incidence of appendicitis^8^. An epidemiologic study in England found that the intake of green vegetables and tomatoes might be a possible protective factor which probably acts through influencing bacterial infections of the appendix^24^. Moreover, a cross-sectional study in the United States reported that many patients with GERD symptoms were accompanied by high dietary fat intake, and that study further found that high fiber intake was correlated with a reduced risk of GERD^25^. Accordingly, dietary habits in patients with GERD could be a potential risk factor for acute appendicitis.

Furthermore, many studies suggested that bacterial infections are considered a very important factor in the occurrence of appendicitis^9^,^26^. For example, one study reported that Fusobacterium infections might play important roles in abdominal infections, including appendicitis, chronic ulcerative colitis, peritonitis, etc^26^. In addition, a previous study showed that local infections with F. nucleatum and F. necrophorum were major reasons for the incidence of acute appendicitis^9^. In addition, a study in Finland showed that the long-term use of PPIs can lead to hypochlorhydria and further induce the intragastric overgrowth of aerobic bacteria including Fusobacterium spp^17^. Consequently, it is plausible that long-term use of PPIs in GERD patients might contribute to the development of appendicitis.

Additionally, this study performed a subgroup analyses to demonstrate the association between prior GERD and appendicitis in different age group because prior studies presented that the incidence and condition of appendicitis varied among the age groups. For instance, the acute appendicitis and non-perforated appendicitis was frequently found in young and middle-age population^2^,^22^. Furthermore, elder population with appendicitis commonly presented non-specific presentation and further contributed to diagnostic difficulty^2^. The findings in this study showed consistent associations between GERD and appendicitis in all age groups, even after adjusting for socioeconomic variables. Age may not affect the relationship between prior GERD and appendicitis.

The specific strength of this case-control study was the use of a population database with widespread health benefit coverage in Taiwan. Characteristics of the LHID2005 can avoid potential selection bias which frequently occurs in observational studies. Moreover, the LHID2005 provides a sufficient sample size and elevated statistical power to detect the relationship between GERD and appendicitis. Nonetheless, several limitations to this study still need to be considered. First, the LHID2005 provides no information on dietary habits, bacterial cultures, or the severity of GERD which are considered to be possible risk factors for appendicitis and might further affect the association between GERD and appendicitis^8^. Second, even though the LHID2005 is a population-based database, it might not include all patients with GERD in Taiwan. Several patients with mild symptoms of GERD might not seek health services covered by the NHI program because they considered the relevant treatments to be unnecessary. Third, the diagnosis of GERD was typically based on the clinical symptoms and endoscopic examination. Therefore, there might be a possibility of selection bias in this study. Finally, most of the patients involved in this study were of Chinese ethnicity. Thus, the ability to generalize the findings of this study to other ethnic groups is not certain.

In conclusion, this population-based case-control study showed that patients with appendicitis have a higher prevalence of prior GERD compared to those without appendicitis. This association remained across all age groups. In clinical aspect, we recommend that physicians should notice the association between GERD and appendicitis and be alert in suspecting appendicitis for abdominal pain in patients with a medical history of GERD. However, more experimental research is still warranted to define the actual underlying mechanisms of the connection between GERD and appendicitis.

## Additional Information

**How to cite this article**: Kao, L.-T. *et al*. Association between Gastroesophageal Reflux Disease and Appendicitis: A Population-Based Case-Control Study. *Sci. Rep.* **6**, 22430; doi: 10.1038/srep22430 (2016).

## Figures and Tables

**Table 1:** Demographic characteristics of patients with appendicitis and controls in Taiwan (*n* = 35,565).

Variable	Patients with appendicitis *n *= 7113		Controls*n *= 28,452		*p* value
	Total no.	Column %	Total no.	Column %	
Age (years)					1.000
18~29	2,162	30.4	8,648	30.4	
30~39	1,612	22.7	6,448	22.7	
40~49	1,288	18.1	5,152	18.1	
50~59	926	13.0	3,704	13.0	
60~69	573	8.1	2,292	8.1	
70~79	380	5.3	1,520	5.3	
≥80	172	2.4	688	2.4	
Sex					1.000
Male	3,367	47.3	13,468	47.3	
Female	3,746	52.7	14,984	52.7	
Monthly income					<0.001
≤NT$15,840	2,836	39.9	11,813	41.5	
NT$15,841~25,000	2,512	35.3	9,136	32.1	
≥NT$25,001	1,765	24.8	7,503	26.4	
Geographical region					
Northern	3,404	47.9	13,782	48.4	0.018
Central	1,604	22.6	6,542	23.0	
Southern	1,911	26.9	7,520	26.4	
Eastern	194	2.7	608	2.1	
Urbanization level					0.492
1 (most urbanized)	2,208	31.0	8,997	31.6	
2	1,992	28.0	8,048	28.3	
3	1,181	16.6	4,766	16.8	
4	955	13.4	3,633	12.8	
5 (least urbanized)	777	10.9	3,008	10.6	
Obesity	122	1.7	501	1.8	0.793

The average exchange rate in 2014 was US$1.00≈New Taiwan Dollar (NT$)30.

**Table 2:** Prevalence, odds ratios (ORs), and 95% confidence intervals (CIs) for gastroesophageal reflux disease (GERD) among sampled patients.

Prior presence of GERD	Total (*n* = 35,565)		Patients with appendicitis (*n* = 7113)		Controls (*n* = 28,452)	
	*n*, %		*n*, %		*n*, %	
Yes	1087	3.06	359	5.05	728	2.56
Crude OR (95% CI)	–		2.04*** (1.79~2.32)		1.00	
Adjusted OR (95% CI)	–		2.05*** (1.80~2.33)		1.00	

Notes: The adjusted OR was calculated by a conditional logistic regression which was adjusted for monthly income and geographic region. ***p < 0.001.

**Table 3:** Prevalence, odds ratios (ORs), and 95% confidence intervals (CIs) for gastroesophageal reflux disease (GERD) among sampled patients according to age group.

Prior presence of GERD	18~39 years old (*n* = 18,870)		40~59 years old (*n* = 11,070)		≥60 years old (*n* = 5625)	
	Patients with appendicitis (*n* = 3774)	Controls (*n* = 15,096)	Patients with appendicitis (*n* = 2214)	Controls (*n* = 8856)	Patients with appendicitis (*n* = 1125)	Controls (*n* = 4500)
	*n*, %	*n*, %	*n*, %	*n*, %	*n*, %	*n*, %
Yes	111 (2.94)	229 (1.52)	163 (7.36)	289 (3.26)	85 (7.56)	210 (4.67)
Crude OR (95% CI)	1.97*** (1.57~2.48)	1.00	2.36*** (1.94~2.88)	1.00	1.67*** (1.29~2.17)	1.00
Adjusted OR (95% CI)	1.96*** (1.56~2.47)	1.00	2.36*** (1.94~2.88)	1.00	1.71*** (1.31~2.22)	1.00

Notes: The adjusted OR was calculated by a conditional logistic regression which was adjusted for monthly income and geographic region. ***p < 0.001.
